# West Nile and Usutu viruses co-circulation in central Italy: outcomes of the 2018 integrated surveillance

**DOI:** 10.1186/s13071-021-04736-z

**Published:** 2021-05-07

**Authors:** Paola Scaramozzino, Andrea Carvelli, Gianpaolo Bruni, Giuseppina Cappiello, Francesco Censi, Adele Magliano, Giuseppe Manna, Ida Ricci, Pasquale Rombolà, Federico Romiti, Francesca Rosone, Marcello Giovanni Sala, Maria Teresa Scicluna, Stefania Vaglio, Claudio De Liberato

**Affiliations:** 1 Istituto Zooprofilattico Sperimentale del Lazio e della Toscana “M. Aleandri”, Via Appia Nuova 1411, 00178 Roma, Italy; 2 Ospedale “S. Pertini”, Via dei Monti Tiburtini 385, 00157 Roma, Italy; 3 Azienda Sanitaria Locale di Latina, Via Pier Luigi Nervi, Latina Fiori, 04100 Latina, Italy; 4 Università degli Studi di Roma “La Sapienza”, Piazzale Aldo Moro 5, 00185 Roma, Italy

**Keywords:** Co-circulation, *Culex pipiens*, Italy, Surveillance, Usutu virus, West Nile virus

## Abstract

**Background:**

West Nile (WNV) and Usutu (USUV) are emerging vector-borne zoonotic flaviviruses. They are antigenically very similar, sharing the same life cycle with birds as amplification host, Culicidae as vector, and man/horse as dead-end host. They can co-circulate in an overlapping geographic range. In Europe, surveillance plans annually detect several outbreaks.

**Methods:**

In Italy, a WNV/USUV surveillance plan is in place through passive and active surveillance. After a 2018 WNV outbreak, a reinforced integrated risk-based surveillance was performed in four municipalities through clinical and serological surveillance in horses, Culicidae catches, and testing on human blood-based products for transfusion.

**Results:**

Eight WNV cases in eight equine holdings were detected. Twenty-three mosquitoe catches were performed and 2367 specimens of *Culex pipiens* caught; 17 pools were USUV positive. A total of 8889 human blood donations were tested, and two asymptomatic donors were USUV positive.

**Conclusions:**

Different surveillance components simultaneously detected WNV only in horses and USUV only in humans and mosquitoes. While in endemic areas (i.e. northern Italy) entomological surveillance is successfully used as an early detection warning, this method in central Italy seems ineffective. To achieve a high level of sensitivity, the entomological trapping effort should probably exceed a reasonable balance between cost and performance. Besides, WNV/USUV early detection can be addressed by horses and birds. Further research is needed to adapt the surveillance components in different epidemiological contexts.

**Graphical Abstract:**

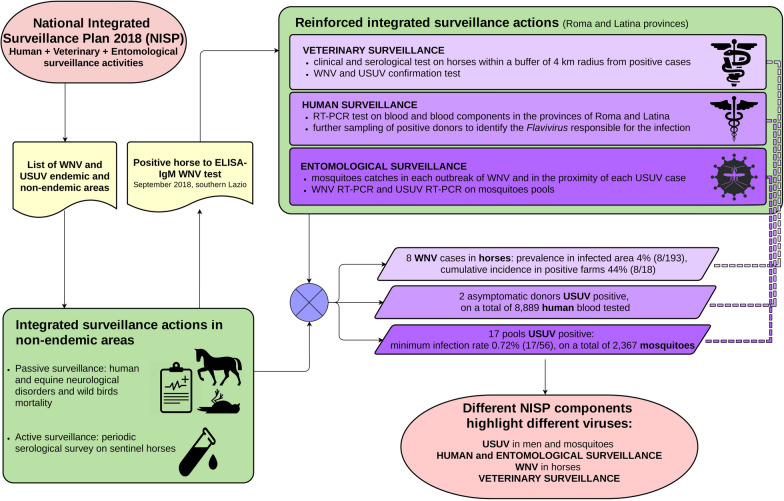

## Background

West Nile (WNV) and Usutu (USUV) viruses are emerging vector-borne flaviviruses within the Flaviviridae family, belonging to the Japanese encephalitis antigenic complex [1]. Spillover from a vertebrate reservoir to humans and other mammals can produce subclinical infections or fatal disease with rare cases of neurological signs [1]. WNV and USUV are genetically and antigenically very similar, sharing the same life cycle, with birds as amplifying hosts and mosquitoes of the genus *Culex* as main vectors. Therefore, WNV and USUV can share the same environment and can often co-circulate in an overlapping geographic range [2].

The first reported outbreaks of WNV in the European area were notified in Romania in 1996 [3]. During the period 2011–2017, 1226 human cases of West Nile disease (WND) were detected in the European Union, mainly in Italy, Greece, Hungary, and Romania [4, 5]. In 2018, a steep rise in the number of reported cases occurred, with 1548 human notified cases, Italy being the most affected country, with 610 cases and 49 fatalities [4].

The first evidence of USUV in Europe was an epidemic among common blackbirds (*Turdus merula*) in Austria in 2001, although a retrospective study showed that it had been present since 1996 in Italy [6, 7]. During the following years, cases in different species were reported from other countries [1, 8–10]. To date, USUV has been detected in 15 countries in Europe and is considered to be spreading [10].

Given the presence of recurrent viral circulation, most of the European countries established surveillance plans with similar epidemiological approaches [11]. The sensitivity of a surveillance system depends on several variables including the susceptibility of the tested population, the diagnostic sensitivity, the prevalence of the infection, and the design of the sampling program.

In Italy, after the first appearance of WNV in 1998 [12], a surveillance plan was established through entomological surveillance, passive clinical surveillance in humans and horses, and active surveillance in horses and targeted synanthropic birds (*Pica pica*,* Garrulus glandarius*,* Corvus corone*). The surveillance plan was able to detect WNV re-emergence in 2008, and it was rescheduled to also detect USUV after its diagnosis in two human patients with neurological disorders in 2009 [13]. Since then, the surveillance for both viruses has beens planned yearly in the National Integrated Surveillance Plan (NISP), based on entomological, veterinary, and human surveillance activities. In 2016, a WNV epidemic occurred in Grosseto and Livorno Provinces, involving several equine holdings distributed over a large area in southern Toscana. During 2017, WNV spread further, detected in horses in northern Lazio (Viterbo Province) and in humans in western Toscana (Livorno Province). In the same year, one asymptomatic human case of USUV (blood donor) was recorded in the Lazio region [14]. Based on the results of the surveillance activities and virus circulation, the 2018 NISP classified some provinces of Lazio and Toscana (Viterbo, Grosseto, Livorno, and Pisa) endemic for WND. In these areas, the surveillance was based on direct testing (real-time RT-PCR) performed on wild captured synanthropic birds (active surveillance) and mosquitoe catches (entomological surveillance). In areas that were not classified endemic, a serological survey on sentinel horses or backyards poultry was also planned, in addition to the activities on synanthropic birds and mosquitoes. Passive clinical surveillance based on human and equine neurological disorders and on anomalous mortalities among wild birds was kept running in the whole territory. Extra mosquitoes sampling was performed where WNV circulation was suspected to have occurred, and all the horses in a buffer of 4 km radius from the suspect case (human or animal) were serologically tested. Overall, in Lazio and Toscana, about 1300 horse and 1200 backyard poultry sera were analyzed for WND antibodies with an ELISA test. Moreover, almost 1000 specific RT-PCR tests for WNV and USUV on synanthropic birds and 450 tests on mosquitoes catches were performed.

None of the above-mentioned activities detected viral circulation during 2018 in the declared endemic territories. Contrarily, in a small territory (southern Lazio) not included in the risk area, WNV and USUV were detected in the late summer-autumn of 2018. Several authors reported the occurrence of co-circulation of WNV and USUV, often as an outcome of the WNV surveillance, since the USUV infection in humans is often asymptomatic [15–17].

The aim of the present article is to describe the co-circulation of WNV and USUV, detected for the first time in central Italy, and to evaluate the sensitivity and the efficacy of the components of surveillance activities.

## Methods

### Integrated surveillance

**Fig. 1:**
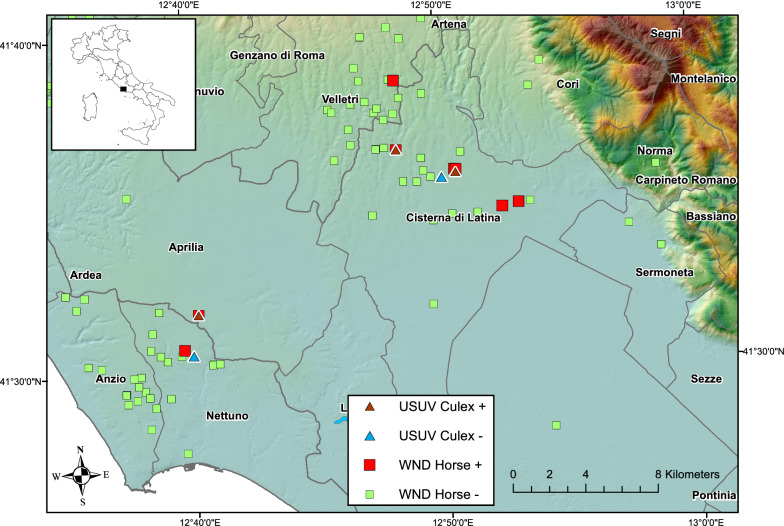
Location of WNV/USUV positive and negative holdings during 2018 surveillance activities in central Italy

The study area was located within the provinces of Roma and Latina and included four municipalities: Aprilia, Cisterna di Latina, Nettuno, and Velletri (512 km^2^) (Fig. 1). On 11 September 2018, a horse in the municipality of Cisterna di Latina tested positive to an IgM WNV ELISA test. As prescribed by the NISP, this was what triggered the start of the reinforced integrated surveillance. The Veterinary Service of the Local Health Unit implemented risk-based, clinical, and serological surveillance in the equine holdings in a 4 km radius from the positive animal, along with specific Culicidae catches in the same holdings. In addition, a mandatory WNV test was performed on human blood and blood components (including hematopoietic stem cells from peripheral, cord and medullary blood), collected in the provinces of Roma and Latina, and on donors who spent at least 1 night in the two provinces, during the 28 days following the last confirmed case.

All sera from WNV-positive animals were sent to the National Reference Centre (NRC) for diagnostic confirmation of WND and USUV. Insect catches were performed in each outbreak, defined as a holding with at least one positive horse. Mosquitoes catches were also performed in the proximity of USUV cases detected in a blood donor. In the study area, no active surveillance of synanthropic birds was performed, and no bird mortality was recorded.

In veterinary surveillance activities, the definition of a WND case was based on NISP: (i) an ELISA IgM-positive test in horses, confirmed by virus neutralization test (VNT); (ii) clinical signs in horses referred to WND and a serological or RT-PCR-positive test; a RT-PCR-positive test in horse blood or tissue or in mosquitoes. A USUV case was defined as a RT-PCR-positive test in horse blood or tissue or in mosquitoes.

In humans, a WND/USUV case was defined as: (1) clinical signs; (ii) virus isolation or RNA identification in blood, urine or cerebrospinal fluid; (iii) presence of antibodies in liquor; (iv) IgM high titer and IgG, confirmed by VNT.

### Field and laboratory activities

Local Health Unit official veterinarians collected serum samples in horses for screening by an Antibody Capture ELISA (MAC) test (ID Screen^®^ West Nile IgM Capture, IDVet, France) for the detection of IgM antibodies against the WNV. This test is specific and demonstrates no cross-reactivity with other flaviviruses, as reported by the producer.

Reactive sera (positive and equivocal) in ELISA were assayed by VNT performed by the NRC. Positive sera were assayed by VNT against WNV and USUV to exclude potential cross-reaction between the two viruses.

One hundred micrograms (µg) of equine brain and medulla oblongata tissues was placed into a 2-ml plastic vial with a 5-mm stainless steel grinding ball and 1 ml Trizol reagent, ground with Tissue Lyser II (Qiagen) and homogenized at 30 Hz for 3 min, followed by centrifugation at 17 × *g* for 10 min at 4 °C.

Mosquitoes catches were performed using CDC, BG Sentinel and Gravid traps, baited respectively with dry ice, BG lure and a mixture of water and hay soiled with guinea pig feces and urine. Sampling protocol (number and frequency of collection of catches) was defined depending on the number of WNV cases in the area and on the number of mosquitoes caught annually in each catch session. After mosquitoes sorting and morphological identification according to Severini et al. [18], *Culex pipiens* females of the same catch were divided in pools containing up to 200 specimens. Mosquitoes were placed into a 2-ml plastic vial with a 5-mm stainless steel grinding ball and 1 ml of Trizol reagent, ground with Tissue Lyser II (Qiagen) and homogenized at 30 Hz for 3 min. The homogenate was then filtered with a 0.45-µm filter to remove the wings and other chitin debris and centrifuged at 17 × *g* for 10 min at 4 °C.

In each pool of mosquitoes and in equine brain and medulla oblongata tissues, 200 µl of the supernatant was used for the nucleic acid extraction using the QIAamp cador Pathogen Mini Kit (Qiagen). A volume of 5 µl from a total volume of 60 µl of eluate was used for the real-time RT-PCR.

Equine blood samples and mosquitoe pools were analyzed by real-time RT-PCR distinctive for WNV Lineage 1 and Lineage 2 and for USUV [19, 20]. The real-time RT-PCR protocols were carried out using the Quant Studio 7 Flex System (Applied Biosystems, Foster City, CA, USA), using the AgPath-ID One-Step RT-PCR Reagents as amplification mix.

Laboratory tests on animal and insect samples were performed by the Public Health Institute ‘Istituto Zooprofilattico Sperimentale del Lazio e della Toscana’ (IZSLT). Human blood samples were analyzed by the Biological Qualification Centre, Sandro Pertini Hospital, using a screening test and WNV nucleic acid amplification test (NAT) (Cobas 6800, Roche, Mannheim, Germany) (modified from [21], amplicon size: 210 nt). The NAT test has a high sensitivity for both WNV lineages but also a broad cross-reactivity to other flaviviruses, in particular, USUV, Kunjin virus, Japanese encephalitis virus, Murray Valley encephalitis virus and Saint Louis encephalitis virus [21]. Plasma aliquots of the donations were sent to the Regional Reference Laboratory for Arboviruses (National Institute for Infectious Diseases ‘L. Spallanzani,’ Roma) for confirmatory testing and further diagnostic investigation. In case of a positive test, the NAT test was repeated twice and the donor was recalled 3 weeks after donation to be sampled again for direct and indirect tests and virus sequencing [22] to detect a possible seroconversion and to identify which flavivirus was involved. Human blood samples were analyzed by anti-USUV Real Time RT-PCR (modified from [23]). Serological investigation was performed with indirect immunofluorescence assay (IFA) (anti-West Nile virus IFA IgM and IgG, Euroimmun, Germany) for WNV and with IFA using home-made glass slides, prepared with a mix of uninfected and USUV-infected Vero E6 cells, according to standard procedures established for flaviviruses and for USUV IgM and IgG antibodies [24].

## Results

Surveillance activities were performed in four municipalities, where 657 equine holdings were present. Eight cases of WND in horses hosted in 8 different equine holdings were detected by direct or indirect methods on a total of 193 tested equids (Fig. 1; Table 1). The overall prevalence was 4% (8/193), while the cumulative incidence in the holdings with at least one positive horse was 8/18 (44%). The last positive case was recorded on 24 October. In particular, none of the seven horses that were positive for the IgM ELISA test were positive on a subsequent RT-PCR test on a whole-blood sample (Table 1). One horse was directly tested by RT PCR on blood because it was classified as a clinical suspect case. After a first negative result, the animal died, and nervous tissues were tested by RT-PCR; the result was WNV lineage 2 positive. All the positive horses were checked for previous vaccinations and had not been vaccinated for WNV, and they were therefore considered confirmed cases, being all positive at ELISA IgG and 5/8 positive at VNT (with titers between 1:5 and 1:80). Only one of the tested horses had low positivity for USUV antibodies (VNT: titer 1:5) (Table 1).

**Table 1:** Diagnostic and clinical findings during WNV/USUV 2018 integrated surveillance in central Italy

Municipality	Farm ID	Species	Case	WNV						USUV	
				Clinical	IgM	IgG	VNT	RT-PCR blood	RT-PCR tissue	PCR	VNT
Cisterna di LT	FARM 1	Horse 1	WND +	+	+	+	+ (1:80)	–		–	–
Cisterna di LT	FARM 1	Horse 2	No	–	–	+	–	–		–	–
Cisterna di LT	FARM 1	Horse 3	No	–	–	+	–	–		–	–
Cisterna di LT	FARM 1	Culex	USUV +						–	+	
Cisterna di LT	FARM 1	Culex	USUV +						–	+	
Cisterna di LT	FARM 1	Culex	USUV +						–	+	
Cisterna di LT	FARM 2	Horse 4	WND +	–	+	+	–	–		–	–
Cisterna di LT	FARM 3	Horse 5	WND +	–	+	+	–	–		–	–
Cisterna di LT	FARM 4	Horse 6	WND +	–	+	+	+ (1:80)	–		–	–
Cisterna di LT	FARM 5	Horse 7	WND +	+	+	+	+ (1:5)	–		–	–
Cisterna di LT	FARM 5	Horse 8	No	–	–	+	–	–		–	+ (1:10)
Cisterna di LT	FARM 10	Culex	USUV +						–	+	
Aprilia	FARM 6	Horse 9	WND +	+	+	+	+ (1:5)	–	+ ^a^	–	–
Aprilia	FARM 6	Culex	USUV +						–	+	
Nettuno	FARM 7	Horse 10	WND +	–	+	+	+ (1:20)	NP		NP	–
Velletri	FARM 8	Horse 11	WND +	–	±	+	+ (1:40)	–		–	–
Velletri	FARM 9	Horse 12	No	–	±	+	+ (1:5)	–		–	–

				WNV						USUV	
				Clinical	IgM	IFA IgG		RT-PCR blood	RT-PCR tissue	PCR	IFA IgM
Cisterna di LT		Human 1	USUV +	–	–	–		+		+	–
Cisterna di LT		Human 2	USUV +	–	–	–		+		+	sc

*IgG* immunoglobulin G, *IgM* immunoglobulin M, *NP* not performed, *RT-PCR* reverse transcriptase-polymerase chain reaction, *VNT* virus neutralization test, *sc* seroconversion (< 1:20 to 1:40 after 3 weeks)

^a^Lineage 2

No anomalous bird mortality was recorded or reported to the Local Health Unit.

Regarding the entomological surveillance, 23 adult mosquitoes catches were performed during the period September–November in eight sites with flavivirus circulation. A total of 2367 specimens of *C. pipiens* were caught, divided into 56 pools and tested by RT-PCR for the detection of WNV and USUV. Among the 56 tested pools, 17 tested positive for USUV, originating from three sites in two municipalities of Latina province (Fig. 1; Table 1). The overall minimum infection rate (MIR) was 0.72%. Considering only the positive sites for USUV circulation, MIRs were, respectively 0.70%, 0.83% and 0.28%. WNV RNA was not detected in any mosquitoes pool.

In 2018, 4611 and 4278 human blood donations were analyzed in Roma and Latina provinces, respectively, with a total of 31,970 in the Lazio region. Two asymptomatic donors tested positive at the WNV NAT screening test and were considered ineligible for the transfusion service. Donors testing USUV RT-PCR positive at the time of donation and sequencing indicated that the USUV strains belonged to clade Europe 2 [22]. One donor was negative to both serological tests while a USUV seroconversion was observed in the second donor (IgM 1:40). Both donors resided in Cisterna di Latina municipality.

## Discussion

This is the first report of WNV and USUV co-circulation in central Italy. Similar findings were reported from northern areas of the country [13, 15, 17]. In Italy, the 2018 summer was exceptionally warm and rainy, probably creating environmental conditions particularly suitable for *C. pipiens* development [25]. This would explain the co-circulation of two vectorborne viruses. Similar findings were also reported from Austria in the same year [26].

It is very interesting to note that in central Italy in 2018, different components of the integrated surveillance system separately detected the two different viruses, even though they were co-circulating. Surveillance activities reported an unexpected epidemiological pattern, the simultaneous report of WNV in horses and of USUV in humans and vectors.

In particular, horse surveillance revealed WNV, but mosquitoes caught at sites where WNV was detected in horses were positive only for USUV (Fig. 1). Furthermore, in the same area where WNV circulation was found in horses, two human blood donors tested positive for USUV, but no anomalous mortality of wild birds was recorded. This event is not often observed, and it might indicate the possible development of herd immunity in wild bird populations (e.g., [27]). The virus circulation among wild birds is frequently detected by active surveillance [14, 17]. Unfortunately, this activity was not carried out in the study area in 2018.

These findings could suggest a different sensitivity of the integrated surveillance components against WNV and USUV in different epidemiological niches. In Italy, different surveillance strategies are planned by the NISP depending on an annual classification of zones as endemic. So far, the experience in the north of the country suggests that entomological surveillance is the best early detection method for virus circulation [28]. In 2018, in northern Italy the first positive pools of *C. pipiens* were detected earlier than usual [14]. In central Italy, the use of entomological surveillance as a tool for early detection seems not to be very effective. In the Lazio region, none of the mosquitoe pools has ever been found positive for WNV in > 10 years of entomological surveillance, while WNV circulation was detected by serological surveillance in horses, whose relevance for public health was already emphasized by Young et al. [29].

The diagnostic tests used in this study (serological and RT-PCR) in mammals and insects seem to be specific enough for each of the two viruses, thus minimizing the possibilities of cross-reactions, frequent among flaviviruses. It is worth noting that a choice of a generic biomolecular test against Flaviviridae, possibly followed by a specific one, has to be evaluated from the economic point of view, taking into account the specific epidemiological background. Therefore, some diagnostic laboratories prefer to use a generic Flaviviridae PCR, followed by a more specific test in case of positivity (e.g. [30]).

The different results coming from different Italian areas could be explained by a higher abundance of both viruses and vectors in the endemic area of northern Italy compared to central Italy. Due to this difference, entomological surveillance might not be sensitive enough to perform as a virus detection early warning in central Italy. To obtain the same sensitivity of endemic areas in northern Italy, in central Italy the entomological trapping effort should probably exceed a reasonable balance between costs and performance. Besides, low WNV circulation rates can be detected by the finding of a positive horse in holdings with many animals and in areas where no human cases are detected. Horses could act as early detection sentinels as they are continuously exposed to mosquitoe bites [29]. In this context, data from 2018 human and entomological surveillances highlighted a circulation of USUV, without inducing clinical signs in birds or humans. In Italy and Germany, USUV was also detected in the blood of donors and healthy lumberjacks, suggesting that USUV infections may be more frequent than believed and even more common than WNV in areas where both viruses circulate [10]. Consequently, the real incidence and distribution of USUV are likely underestimated, and therefore the public health impact has to be evaluated [31].

## Conclusions

The present study confirms the efficacy of the integrated surveillance to detect both WNV and USUV so far circulating in Italy. In particular, in the same area, horse surveillance revealed WNV, while entomological surveillance and testing of human blood donors revealed USUV. No anomalous birds mortality was recorded. Further research is needed to better adapt the different WNV/USUV surveillance components in different contexts.

## Data Availability

The datasets generated and analyzed during the current study are not publicly available due privacy concerns, but are available from the corresponding author on reasonable request.

## Abbreviations

WNV: West Nile virus
USUV: Usutu virus
*C. pipiens*: *Culex pipiens*
WND: West Nile Disease
NISP: National Integrated Surveillance Plan
RT-PCR: Reverse transcriptase-polymerase chain reaction
NRC: National Reference Centre
MAC: Antibody capture ELISA
VNT: Virus neutralization test
IZSLT: Istituto Zooprofilattico Sperimentale del Lazio e della Toscana
MIR: Minimum infection rate
